# Changes in physical fitness and nutritional status of schoolchildren in a period of 30 years (1980-2010)

**DOI:** 10.1016/j.rpped.2015.03.008

**Published:** 2015

**Authors:** Gerson Luis de Moraes Ferrari, Victor Keihan Rodrigues Matsudo, Mauro Fisberg

**Affiliations:** a Centro de Estudos do Laboratório de Aptidão Física de São Caetano do Sul (CELAFISCS), São Caetano do Sul, SP, Brazil; b Adolescent Medicine Section (Centro de Atendimento e Apoio ao Adolescente - CAAA) of the Department of Pediatrics, Universidade Federal de São Paulo - Escola Paulista de Medicina (Unifesp/EPM), São Paulo, SP, Brazil

**Keywords:** Child, Physical fitness, Nutritional status

## Abstract

**Objective:**

To analyze and compare the changes in physical fitness according to the nutritional status and gender of schoolchildren during a period of 30 years (1980-2010).

**Methods:**

Four cross-sectional evaluations were performed every 10 years in a period of 30 years from 1978 to 1980 (baseline), 1988-1990 (10 years), 1998-2000 (20 years) and 2008-2010 (30 years). The sample consisted of 1291 schoolchildren (188 in baseline, 307 in 10 years; 375 in 20 years; 421 in 30 years) of 10 and 11 years old. The variables assessed were: body weight (kg), height (cm), upper limb strength (ULS; kg), lower limb strength (LLS; cm), agility (seconds) and velocity (seconds). Schoolchildren were classified as normal weight and overweight according to World Health Organization reference of body mass index for age and gender. Comparisons among periods applied ANOVA followed by Bonferroni test, with a significance level set at of *p*<0.01. Variation between baseline and 30 years was assessed by the percentage delta. Seven different percentile values were presented for each variable.

**Results:**

In eutrophic boys and girls, mean values of ULS (−16.7%; −3.2%), agility (−1.5%; −1.6%) decreased significantly after 30 years (*p*<0.001). In the overweight boys and girls, only the average ULS (−15.5%; −12.5%) decreased significantly over time (*p*<0.001). After 30 years, the ULS percentile changed in boys.

**Conclusions:**

The decline in physical fitness was greater in schoolchildren with normal weight than in those with overweight.

## Introduction

Physical fitness can be represented by several variables, and among them are body composition measurements (body weight and body mass index - BMI), as well as neuromotor (muscle strength, agility and velocity) and metabolic variables (cardiorespiratory fitness).^1^^,^ 2 If, on the one hand, the prevalence of overweight schoolchildren has increased alarmingly worldwide,^3^ on the other hand, some physical fitness variables - for instance, muscle strength and cardiorespiratory fitness - have decreased in recent years in several countries.^4^^,^ 5 Some studies have shown that muscle strength and cardiorespiratory fitness are negatively associated with risk factors for cardiovascular disease (CVD).^6^^-^ 8 For instance, overweight schoolchildren with low physical fitness have a higher risk of developing cardiovascular diseases, such as obesity, diabetes and arterial hypertension.^6^^,^ 8

According to Tomkinson,^9^ studies analyzing physical fitness variables over the years, available in developing countries, do not allow firm conclusions to be drawn about the magnitude of possible trends, and temporal studies can provide information on health indicators (such as overweight and physical fitness variables), as well as an understanding of social, economic and cultural changes of a given region. Of all the studies that analyzed the behavior of physical fitness variables over the years in Brazil, only three considered the body size,^10^^-^ 12 as overweight or obese schoolchildren have lower levels of physical fitness when compared to those with normal weight.^13^ We recently showed a greater reduction in cardiorespiratory fitness in schoolchildren classified as having normal weight (25.8%) than in those classified as overweight (16.2%) in a 30-year period.^10^ For this reason, it is suggested that the levels of physical fitness variables may have decreased in recent years at different magnitudes in schoolchildren with different nutritional statuses.^14^ There has been no study in the literature that assessed changes in physical fitness variables involving muscle strength, agility and velocity in schoolchildren of different body sizes over a 30-year period.

In this study, the hypothesis is that the decrease in physical fitness variables (upper and lower-limb strength, agility and velocity), analyzed during a 30-year period, is occurring in both schoolchildren with normal weight and overweight ones. Therefore, the aim of the study was to analyze and compare the changes in physical fitness variables according to the nutritional status and gender in schoolchildren over a 30-year period (1980-2010).

## Method

This study is a cross-sectional analysis, which is part of the Mixed Longitudinal Project on Growth, Development and Physical Fitness of the municipality of Ilhabela, coordinated by the Centro de Estudos do Laboratório de Aptidão Física of São Caetano do Sul (CELAFISCS).^2^ Considering all schoolchildren participating in the study, the number of students with normal weight (*n*=789; 61%) was higher than that of overweight ones (*n*=502). Of the 726 boys, 501 (69%) had normal weight and 225 (31%) were overweight. Of the 565 girls, 288 (51%) had normal weight and 277 (49%) were overweight.^10^ Sampling procedures and inclusion criteria (to have undergone a complete physical examination in one of the analyzed periods; to be between 10 and 11 years old; with no clinical or functional limitations; to be classified as prepubertal, and parents/guardians signing the informed consent form) were published previously.^10^ To determine the biological maturation stage, the self-assessment technique of secondary sexual characteristics according to gender was used, which had already been validated (from 0.60 to 0.71) for the Ilhabela Project itself.^2^ The project was approved by the Institutional Review Board of Universidade Federal de São Paulo (protocol n. 0056/10).

A database containing physical assessments from 1978 to 2011 was analyzed, consisting of 1291 students (Table 1) aged 10 and 11 years who participated in the study. Schoolchildren in this age group were selected in each of the four periods with cross-sectional analyses, carried out every 10 years and over a period of 30 years, from 1978 to 1980 (baseline), 1988-1990 (10 years), 1998-2000 (20 years) and 2008-2010 (30 years).

**Table 1:** Descriptive analysis of anthropometric variables of schoolchildren from Ilhabela over a 30-year period.

	1978–1980	1988–1990	1998–2000	2008–2010
*Gender* a				
Male	93 (49.5)	173 (56.3)	212 (56.5)	248 (58.9)
Female	95 (50.5)	134 (43.7)	163 (43.5)	173 (41.1)

*Nutritional status* a				
Normal weight	94 (50.0)	172 (56.0)	248 (66.2)	275 (65.4)
Excess weight	94 (50.0)	135 (44.0)	127 (33.8)	146 (34.6)
Body weight (kg)^b^	36.22±8.30	35.47±8.03	36.24±8.34	35.90±7.46
Height (cm)^b^	142.83±7.98	141.20±8.71	142.65±7.55	143.06±7.83
BMI (kg/m^2^)^b^	17.56±2.2	17.61±2.7	17.65±2.2	17.41±2.1

BMI, body mass index.

a Data described as number (percentage).

b Data described as mean ± standard deviation.

All variables included in this study were evaluated in the four periods, according to the CELAFISCS standardization.^2^ Of the anthropometric variables, body weight and height were measured.^2^ Three consecutive measurements were obtained, and the arithmetic mean was used for the analysis. BMI (kg/m^2^) was used for the classification of nutritional status. To meet the objective of the present study, children were classified into two groups: normal weight, when the *z*-score was between −2 and 1, and overweight, when *z* score was >1, according to the BMI curves proposed by the World Health Organization (WHO).^15^Schoolchildren with *z*-score <−2 were excluded from the study.

Physical fitness variables that were analyzed included upper (ULS) and lower-limb strength (LLS), agility and velocity.^2^ ULS was measured by a handgrip dynamometer (Takei TK 005, Tokyo, Japan) in kg. The students gripped the dynamometer with the greatest possible strength with the right hand, with the arm extended along the body. The best result of two attempts, performed at least two minutes apart, was considered the final result. LLS measurement was obtained by the vertical jump test without help from the upper limbs, measured in cm. The tests were performed three times, and the best result was considered for the analysis. Agility was measured by the shuttle run test, and the best result of two attempts was considered. Velocity was evaluated using the 50-m run test in a single attempt. Before each test, the test objectives and procedures were briefly explained to facilitate their understanding by the schoolchildren.

The reproducibility and objectivity of each test were calculated in a sub-sample of 40 students in each evaluation. The reproducibility values ranged from 0.95 to 0.97 for body weight, 0.97-0.99 for height, 0.74 to 0.77 for upper-limb strength, from 0.77 to 0.81 for lower-limb strength, from 0.76 to 0.79 for agility, and 0.77 to 0.81 for velocity. Regarding the objectivity, the values were 0.94-0.98 for body weight, 0.96 to 0.99 for height, 0.75 to 0.79 for upper-limb strength, 0.78 to 0.82 for lower-limb strength, 0.77 to 0.80 for agility, and 0.79 to 0.83 for velocity.

Data distribution was analyzed using the Kolmogorov-Smirnov test, and the variables were expressed as mean and standard deviation for numerical variables. The variation between baseline and 30 years was carried out using the delta percentage (∆%). The comparison of the four assessments was performed by analysis of variance (ANOVA) with three factors (gender, nutritional status and decade), followed by Bonferroni multiple comparison test.^16^ Seven different percentile values (5, 10, 25, 50, 75, 90 and 95) were calculated for each decade, analyzed by gender for each of the variables analyzed. The calculations were performed using the Statistical Package for the Social Sciences software (SPSS) version 18.0, and statistical significance was set at *p*<0.01.^16^

## Results

The proportion of male schoolchildren classified by nutritional status according to the decade was: 1978-1980: 27.2% normal weight and 22.3% overweight; 1988-1990: 35.8% normal weight and 20.5% overweight; 1998-2000: 41.6% normal weight and 14.9% overweight; 2008-2010: 43.8% normal weight and 15.2% overweight. The number of male schoolchildren with normal weight was higher than those with overweight in all evaluations. As for females, the proportion was: 1978-1980, baseline: 22.8% with normal weight and 27.7% overweight; 1988-1990: 20.2% with normal weight and 23.5% overweight; 1998-2000: 24.6% normal weight and 18.9% overweight; 2008-2010: 21.6% normal weight and 19.4% overweight. The number of female schoolchildren with normal weight was higher only in the 20 and 30-year evaluations.^10^ Table 1 shows the characterization (gender, nutritional status and anthropometry) of the sample according to the four evaluation periods.

In boys with normal weight, mean upper-limb strength values decreased significantly after 30 years. As for agility, the average means when comparing the 2008-2010 and the 1978-1980 periods increased. There were no significant differences between the periods regarding lower-limb strength and velocity (Table 2). In overweight boys, upper-limb strength showed a statistically higher mean in 1978-1980, when compared with 2008-2010. There were no significant differences between the evaluation periods of lower-limb strength, agility and velocity (Table 2).

**Table 2:** Comparison of physical fitness variables according to gender and nutritional status of schoolchildren from Ilhabela over a 30-year period.

	1978–1980	1988–1990	1998–2000	2008–2010	*p* a	Δ%^b^
***Normal weight***						
*Gender*						
Male	*n*=51	*n*=110	*n*=156	*n*=184		
Female	*n*=43	*n*=62	*n*=92	*n*=91		

*ULS (kg)*						
Male	21.50±6.92^c^	19.02±4.25^c^	18.06±4.55^c^	17.92±3.65	<0.001	−16.7
Female	18.74±4.88	18.72±4.05	16.18±3.60^c^^,^ d	18.16±4.12	<0.001	−3.2

*LLS (cm)*						
Male	24.60±4.64	24.55±4.82	24.47±4.99	22.38±5.92	0.40	−9.0
Female	24.41±3.86	24.41±4.41	23.51±4.76	22.02±4.89	0.47	−9.8

*Agility (seconds)*						
Male	12.54±1.28^c^	12.66±1.19	12.69±1.01	12.73±1.07	<0.001	−1.5
Female	13.51±1.27^c^^,^ d	13.71±1.17^d^	12.93±0.84^d^	13.72±1.60^d^	<0.001	−1.6

*Velocity (seconds)*						
Male	9.49±0.73	9.70±0.83	9.88±0.86	10.10±2.37	0.04	−6.4
Female	9.59±0.66	10.36±0.87^d^	10.36±0.72	10.32±0.83	0.89	−7.0

***Excess weight***						
*Gender*						
Male	*n*=42	*n*=63	*n*=56	*n*=64		
Female	*n*=52	*n*=72	*n*=71	*n*=82		

*ULS (kg)*						
Male	23.75±6.18^c^	20.83±4.24^e^	18.69±5.20^e^	20.07±4.33^e^	<0.001	−15.5
Female	22.60±5.17	20.56±4.96^c^^,^ e	15.73±3.86^c^^,^ d ^,^ e	20.08±4.35^e^	<0.001	−12.5

*LLS (cm)*						
Male	22.31±4.62	24.10±6.04	22.60±5.90	24.58±4.75	0.27	10.2
Female	24.75±5.44	23.09±4.58	21.32±4.32	23.10±5.42	0.14	−6.7

*Agility (seconds)*						
Male	13.15±1.02	13.04±1.14	12.82±1.00	13.06±0.89	0.54	0.7
Female	13.86±1.40^d^	13.83±1.24^d^	13.13±1.05	13.66±0.97	0.04	1.4

*Velocity (seconds)*						
Male	10.00±0.68	10.05±0.98	10.53±1.20^e^	10.43±1.00	0.13	−4.3
Female	10.10±0.40	10.60±0.82	11.06±1.14^e^	10.92±1.08	0.17	−7.5

ULS, upper-limb strength; LLS, lower-limb strength; data described as mean ± standard deviation.

a ANOVA with three factors (gender, nutritional status and decade).

b Δ% (delta percentile): variation between the baseline and 30 years.

c *p*<0.01: for comparison between baseline, 10, 20, and 30 years.

d *p*<0.01: for comparison between boys and girls.

e *p*<0.01: for comparison between normal and overweight individuals.

In normal weight girls, upper-limb strength showed higher means when comparing the 1998-2000 and the 2008-2010 periods. The means of agility times increased when comparing 2008-2010 vs. 1978-1980. Both lower-limb strength and velocity did not differ between evaluation periods (Table 2). In overweight girls, only the mean upper-limb strength decreased statistically when comparing the periods 1988-1990 vs. 2008-2010 and 1998-2000 vs. 2008-2010 (Table 2).

Regarding upper-limb strength, overweight boys had a better performance than the ones with normal weight in the periods 1988-1990, 1998-2000 and 2008-2010. As for velocity, boys with normal weight had a significantly better performance than overweight ones only in the 1998-2000 periods. Normal-weight and overweight boys had similar results regarding lower-limb strength and agility in all analyzed periods (Table 2).

Overweight girls had a better performance regarding upper-limb strength, when compared to normal weight ones, in the 1988-1990, 1998-2000 and 2008-2010 periods. As for velocity, normal weight girls had a significantly better performance than overweight ones only in the 1998-2000 period (Table 2).

In terms of comparison between the genders regarding upper-limb strength, normal weight and overweight girls performed worse than normal-weight and overweight boys only in the period of 1998-2000. Regarding agility, normal-weight girls performed worse than normal-weight boys in all evaluated periods. When overweight girls were compared to overweight boys, the difference between the means occurred only in the 1978-1980 and the 1988-1990 periods. Regarding the lower-limb strength and velocity means, there was no statistical difference between the genders (Table 2).

**Figure 1:**
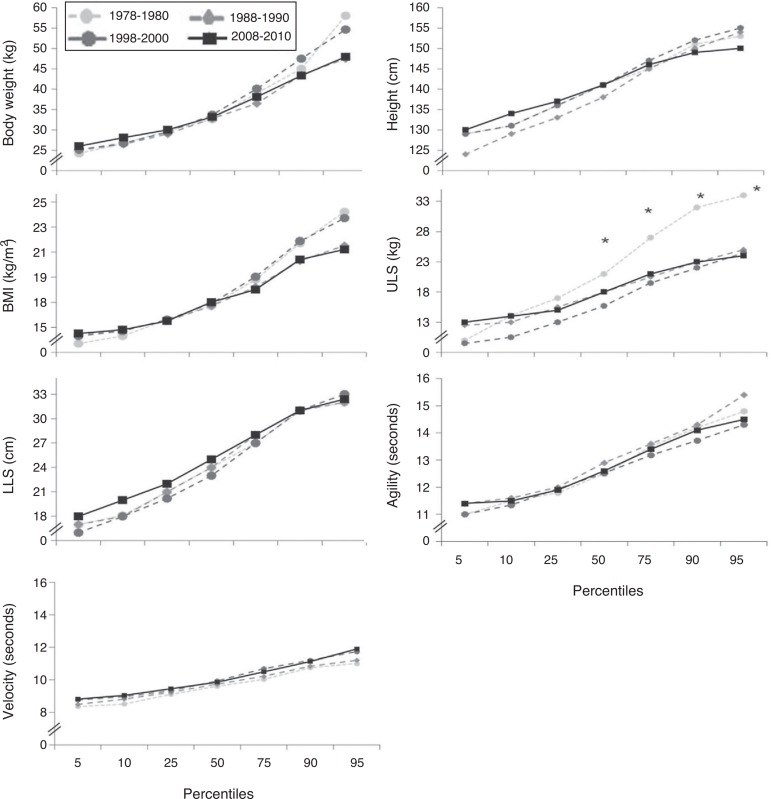
Percentile values for each physical fitness variable regardless of nutritional status over a 30-year period in males. ULS, upper-limb strength; LLS, lower-limb strength; *p<0.01: for comparisons between baseline and 10, 20 and 30 years.

**Figure 2:**
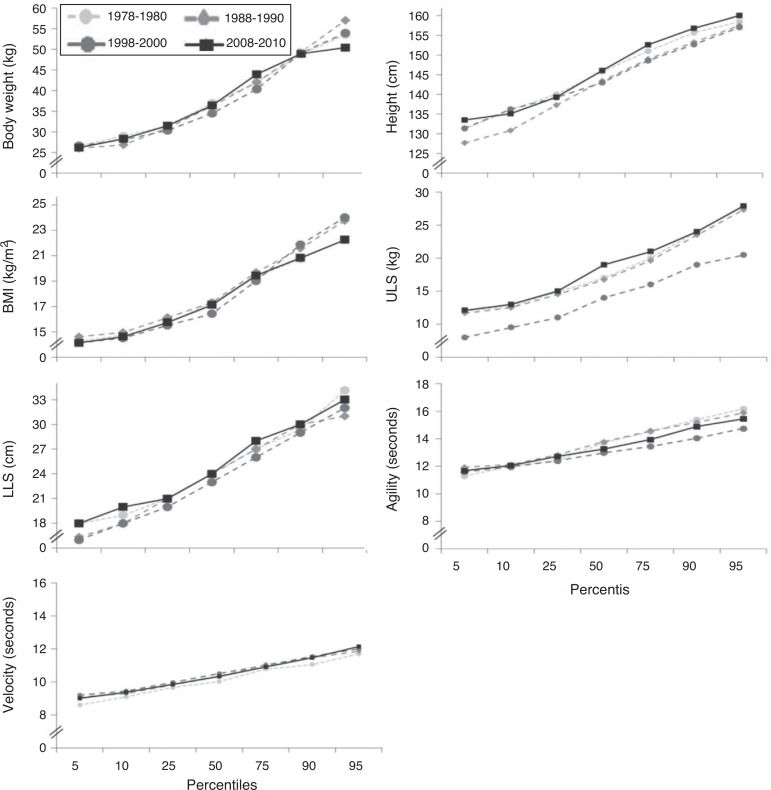
Percentile values for each physical fitness variable regardless of nutritional status over a 30-year-period in females. ULS, upper-limb strength; LLS, lower-limb strength.

Regardless of the nutritional status, Figs. 1(males) and 2 (females) illustrate the percentile values for each physical fitness variable and their respective changes over the years. In males, the mean values of the percentiles (50, 75, 90 and 95) of upper-limb strength decreased significantly when comparing 1978-1980 with 1988-1990, 1998-2000 and 2008-2010 (Fig. 1). In females, there were no significant differences in percentiles between the assessment periods for all analyzed variables (Fig. 2).

## Discussion

In normal children of both genders, there was a significant decrease in the mean upper-limb strength and agility time. In overweight children of both genders, there was a significant decrease only in upper-limb strength over 30 years. These results seem to indicate that physical fitness indicators of schoolchildren from Ilhabela worsened in recent years, although there was no evidence of an increase in childhood obesity in this specific population over the years, unlike the nutritional change, from malnutrition to overweight, which has affected the Brazilian population in the same period during which our data were collected.^17^ Although Ilhabela has shown remarkable economic development in recent decades, with an increase in population and urbanization rates, it is important to note that the nutritional status remained unchanged among the children studied in this age group. Therefore, it seems probable that the schoolchildren observed in this study did not follow the nutrition transition profile of Brazilian schoolchildren in the same studied period. The Household Budget Survey^17^ showed a more than three-fold increase, from 9.8% to 33.4%, in the proportion of Brazilian children with excess weight between 1975 and 2009.

Corroborating the findings of this study, Dollman et al.^18^ found an annual decline in physical fitness in schoolchildren in Australia. The reduction in physical fitness was observed not only in overweight children, but also in those with normal weight.^19^ Our results showed worse performance of overweight students than of the normal weight ones only with respect to velocity. On the other hand, the overweight ones had a better performance in upper-limb strength. Kim et al.^20^ analyzed schoolchildren in the United States and observed worse physical fitness results in obese children than in the normal weight ones in running tests. In both genders, our results did not find changes in lower-limb strength and agility, when comparing normal-weight schoolchildren with overweight ones. For instance, Dollman et al.^18^ found an increase in the 50-meter running time of 0.1-0.2% per year for 12 years. Our results found a reduction in velocity of 0.24% and 0.20% per year in normal weight and overweight schoolchildren, respectively.

Changes in physical fitness over the years have been attributed largely to changes in physical activity.^21^ A study carried out by Artero et al.^22^ indicated that overweight children can perform physical fitness tests as well or even better than those with normal weight, and in this study this trend was found in the upper-limb strength test, especially among boys.

Other results showed that schoolchildren with better physical fitness are less likely to be overweight than those with poorer physical fitness performance.^13^^,^ 23 For instance, Shang et al.^23^disclosed that overweight children had a worse physical fitness performance when compared to normal-weight ones. These results are comparable to those of a cross-sectional study carried out with adolescents from the Republic of Seychelles by Bovet et al.,^13^ which reported an inverse association between physical fitness and body weight.

Overweight schoolchildren are more sedentary and perform less moderate-to-vigorous physical activity than normal-weight ones.^24^A systematic review established a high correlation between lack of physical activity and unfavorable body composition and low physical fitness. All 232 reviewed studies associated a sedentary lifestyle with risk factors for CVD and increased health risks.^25^

According to the model established by Tomkinson and Olds,^5^ factors associated with secular changes are caused by a set of social, behavioral, physical, psychosocial and physiological factors. These patterns can be explained by higher calorie intake rates and reduced energy consumption, associated with better access to technology.

Analyzing the data according to the percentile, decade after decade, there were considerable variations and reductions in physical fitness values, especially in the upper-limb strength of schoolchildren. For instance, observing the 95th percentile for the male gender, upper-limb strength was 33 kg in 1978-1980 and 24 kg 30 years later. Such changes have also been reported in another study.^26^

Regarding gender, the data show that the girls had a worse performance than boys. Research carried out over the years in developed countries (Spain and The Netherlands) showed results similar to those found here regarding both physical fitness and activity.^27^^,^ 28

Body composition, physical fitness and physical activity levels are strongly associated with cardiovascular disease and mortality.^25^^,^ 29 Therefore, the identification of risk groups is crucial for the development of intervention strategies. Changes in lifestyle and the regular practice of physical activity through parental initiatives and social support interventions are important strategies to fight against childhood obesity and physical inactivity.^30^ However, other prospective studies are needed to determine the cause-and-effect relation between physical activity, physical fitness and body weight, as some authors found an inverse association between body weight and physical fitness.^13^

The cross-sectional analyses of this study do not allow us to establish a cause-and-effect association. The convenience sample may have selected children who were more interested in performing the physical fitness tests, limiting the generalization of results. Nevertheless, these limitations do not conceal a trend of decreased physical fitness variables that affect schoolchildren in developing countries, and the results may apply to Brazilian children with the same characteristics. However, they must be interpreted with caution, even if the international literature found decreased physical fitness variables over the years in representative samples, a fact that differs from the current study.^18^^,^ 21 On the other hand, this is the first Brazilian study to show the behavior of neuromotor variables according to the nutritional status of schoolchildren over a 30-year period.

The present study showed that the physical fitness of schoolchildren aged between 10 and 11 years, of both genders, showed a significant reduction over a 30-year period. The decline in physical fitness patterns was higher in normal weight children (upper-limb strength and agility) than in overweight ones. This study emphasizes the complexity of physical fitness patterns of schoolchildren and their development over time, as the nutritional status may have a distinct impact on this outcome over the years. Other research strategies should be explored to better explain physical fitness and its association with nutritional status in children over the years.
