# Supplementary material for: Changes in physical fitness and nutritional status of schoolchildren in a period of 30 years (1980-2010)
Source: Rev Paul Pediatr. 2015 Oct-Dec;33(4):415–22. doi: 10.1016/j.rpped.2015.03.008 (PMC4685561; doi:10.1016/j.rpped.2015.03.008)
Supplement: Translation

# Translation: Modificações da aptidão física e estado nutricional de escolares em um período de 30 anos (1980-2010)

**Authors:** Gerson Luis de Moraes Ferrari, Victor Keihan Rodrigues Matsudo, Mauro Fisberg

**Affiliations:** a Centro de Estudos do Laboratório de Aptidão Física de São Caetano do Sul (Celafiscs), São Caetano do Sul, SP, Brasil; b Setor de Medicina do Adolescente (Centro de Atendimento e Apoio ao Adolescente [CAAA]), Departamento de Pediatria da Universidade Federal de São Paulo, Escola Paulista de Medicina (Unifesp/EPM), São Paulo, SP, Brasil

**Keywords:** Criança, Aptidão física, Estado nutricional

## Abstract

**Objetivo:**

Analisar e comparar as mudanças da aptidão física de acordo com o estado nutricional e sexo de escolares durante 30 anos (1980-2010).

**Métodos:**

Quatro avaliações transversais foram feitas a cada 10 anos em 30 anos: 1978-1980 (linha de base), 1988-1990 (10 anos), 1998-2000 (20 anos) e 2008-2010 (30 anos). A amostra consistiu de 1.291 escolares (188 na linha de base; 307 em 10 anos; 375 em 20 anos; 421 em 30 anos) de 10 e 11 anos. Foram analisados: peso corporal (kg), estatura (cm), força de membros superiores (FMS; kg) e inferiores (FMI; cm), agilidade (segundos) e velocidade (segundos). Os escolares foram classificados como eutróficos e excesso de peso mediante as curvas propostas pela Organização Mundial da Saúde de índice de massa corporal. Foi usado Anova seguida pelo método de Bonferroni para comparar os períodos e adotado *p*<0,01. Variação entre a linha de base e 30 anos foi avaliada pelo delta percentual. Foram apresentados sete distintos valores de percentis para cada variável analisada.

**Resultados:**

Nos meninos e nas meninas eutróficos, as médias dos valores da FMS (-16,7%;-3,2%) e agilidade (-1,5%; -1,6%) diminuíram significativamente em 30 anos (*p*<0,001). Nos meninos e nas meninas com excesso de peso, somente as médias da FMS (-15,5%; -12,5%) diminuíram significativamente (*p*<0,001). Após 30 anos, os valores dos percentis da FMS dos meninos apresentaram diminuição.

**Conclusões:**

Em 30 anos, o declínio da aptidão física foi maior nos escolares eutróficos do que naqueles com excesso de peso.

## Introdução

A aptidão física pode ser representada por diversas variáveis. Entre elas estão medidas de composição corporal (peso corporal e índice de massa corporal [IMC]), variáveis neuromotoras (força muscular, agilidade e velocidade) e metabólicas (aptidão cardiorrespiratória).^1^^,^ 2 Se, por um lado, a prevalência de escolares com excesso de peso tem aumentado de forma alarmante no mundo,^3^ por outro algumas variáveis de aptidão física, por exemplo, a força muscular e a aptidão cardiorrespiratória, têm diminuído nos últimos anos em vários países.^4^^,^ 5 Alguns estudos mostram que a força muscular e a aptidão cardiorrespiratória se associam de forma negativa com fatores de risco para doenças cardiovasculares (DCV).^6^^-^ 8 Por exemplo, escolares com excesso de peso e baixa aptidão física têm maior risco de desenvolver DCV, tais como obesidade, diabetes e hipertensão arterial.^6^^,^ 8

Segundo Tomkinson,^9^ estudos que analisaram as variáveis de aptidão física ao longo dos anos, disponíveis nos países em desenvolvimento, não permitem conclusões consistentes sobre a magnitude de possíveis tendências e estudos temporais podem fornecer informações de indicadores de saúde (como excesso de peso e variáveis de aptidão física) e uma compreensão das mudanças sociais, econômicas e culturais de uma determinada região. De todas as pesquisas que analisaram o comportamento das variáveis de aptidão física ao longo dos anos no Brasil, somente três consideraram o tamanho corporal,^10^^-^ 12 uma vez que escolares com excesso de peso ou obesidade têm baixos níveis de aptidão física em comparação com os eutróficos.^13^ Recentemente, mostramos diminuição maior da aptidão cardiorrespiratória em escolares classificados como eutróficos (25,8%) do que naqueles classificados como portadores de excesso de peso (16,2%) em 30 anos.^10^ Por esse motivo, sugere-se que os níveis das variáveis de aptidão física podem ter diminuído nos últimos anos em magnitudes distintas em escolares de diferentes estados nutricionais.^14^ Não se encontrou na literatura estudo que tenha analisado as alterações das variáveis de aptidão física que envolvem força muscular, agilidade e velocidade em crianças de diferentes tamanhos corporais ao longo de 30 anos.

Neste estudo, a hipótese é que a diminuição das variáveis de aptidão física (força de membros superiores e inferiores, agilidade e velocidade), analisada em 30 anos, ocorra tanto em escolares eutróficos quanto nos escolares com excesso de peso. Com isso, o objetivo do estudo foi analisar e comparar as mudanças das variáveis de aptidão física de acordo com o estado nutricional e sexo entre escolares ao longo de 30 anos (1980-2010).

## Método

Trata-se de um estudo com avaliações transversais que faz parte do Projeto Misto-Longitudinal de Crescimento, Desenvolvimento e Aptidão Física de Ilhabela, coordenado pelo Centro de Estudos do Laboratório de Aptidão Física de São Caetano do Sul (Celafiscs).^2^ De todos os escolares participantes da pesquisa, a quantidade de escolares eutróficos (n=789; 61%) foi maior do que a das portadoras de excesso de peso (n=502). Dos 726 meninos, 501 (69%) eram eutróficos e 225 (31%) tinham excesso de peso. Já nas 565 meninas, 288 (51%) eram eutróficas e 277 (49%) tinham excesso de peso.^10^ Os procedimentos de amostragem e critérios de inclusão (ter uma avaliação física completa em um dos períodos analisados; ter entre 10 e 11 anos; não ter limitações clínicas nem funcionais; ser classificado como pré-púbere e os responsáveis terem assinado o Termo de Consentimento Livre e Esclarecido) foram publicados anteriormente.^10^ Para determinar o estágio de maturação biológica, foi usada a técnica de autoavaliação das características sexuais secundárias de acordo com o sexo, já validada (0,60 a 0,71) no próprio Projeto de Ilhabela.^2^ O projeto foi aprovado pelo Comitê de Ética da Universidade Federal de São Paulo (protocolo 0056/10).

Foi analisado um banco de dados com avaliações físicas de 1978 a 2011; 1.291 escolares (Tabela 1) de 10 e 11 anos participaram do estudo. Foram selecionados escolares nessa faixa etária em cada um dos quatro períodos com avaliações transversais, intercaladas a cada 10 anos e feitas durante 30 anos: 1978-1980 (linha de base), 1988-1990 (10 anos), 1998-2000 (20 anos) e 2008 -2010 (30 anos).

**Tabela 1:** Análise descritiva das variáveis antropométricas dos escolares de Ilhabela em 30 anos

	1978‐1980	1988‐1990	1998‐2000	2008 ‐2010
*Sexo* a				
Masculino	93 (49,5)	173 (56,3)	212 (56,5)	248 (58,9)
Feminino	95 (50,5)	134 (43,7)	163 (43,5)	173 (41,1)
*Estado nutricional* a				
Eutróficos	94 (50,0)	172 (56,0)	248 (66,2)	275 (65,4)
Excesso de peso	94 (50,0)	135 (44,0)	127 (33,8)	146 (34,6)
Peso corporal (kg)^b^	36,22±8,30	35,47±8,03	36,24±8,34	35,90±7,46
Estatura (cm)^b^	142,83±7,98	141,20±8,71	142,65±7,55	143,06±7,83
IMC (kg/m^2^)^b^	17,56±2,52	17,61±2,47	17,65±2,92	17,41±2,51

IMC, índice de massa corporal.

a Dados descritos em número (porcentagem).

b Dados descritos em média ± desvio padrão.

Todas as variáveis incluídas no presente estudo foram avaliadas nos quatro períodos, de acordo com a padronização Celafiscs.^2^ Das variáveis antropométricas, foram mensurados o peso corporal e a estatura.^2^ Três medidas consecutivas foram obtidas e a média aritmética foi usada para análise. Para a classificação do estado nutricional, empregou-se o IMC (kg/m^2^). Para atender ao objetivo do presente estudo, os escolares foram classificados em dois grupos: eutrofia, quando o z-score encontrava-se entre -2 e 1, e excesso de peso, quando o z-escore >1, de acordo com as curvas de IMC proposta pela Organização Mundial da Saúde (OMS).^15^ Escolares com um z-escore <-2 foram excluídos.

As variáveis de aptidão física analisadas foram: força de membros superiores e inferiores, agilidade e velocidade.^2^ A força de membros superiores foi medida por um dinamômetro de preensão manual (Takei TK005, Tóquio, Japão) em kg. Os escolares pressionaram o dinamômetro com a maior força possível com a mão direita e com o braço estendido ao longo do corpo. O melhor resultado de duas tentativas, com pelo menos dois minutos de intervalo, foi considerado como resultado final. A força de membros inferiores foi obtida pelo teste de impulsão vertical sem auxílio dos membros superiores e mensurada em cm. Os testes foram feitos três vezes e o melhor resultado foi considerado para a análise. A agilidade foi feita pelo teste *shuttle run* e foi considerado o melhor resultado de duas tentativas. A avaliação da velocidade foi avaliada por meio do teste de corrida de 50 metros em uma única tentativa. Antes de cada teste, explicaram-se brevemente os objetivos e procedimentos para facilitar a compreensão.

A reprodutibilidade e a objetividade de cada teste foram calculadas em uma subamostra de 40 escolares em cada avaliação. Os valores de reprodutibilidade variaram de 0,95-0,97 para peso corporal, 0,97-0,99 para estatura, 0,74-0,77 para força de membros superiores, 0,77-0,81 para força de membros inferiores, 0,76-0,79 para agilidade e 0,77-0,81 para velocidade. Referente à objetividade, os valores foram de 0,94-0,98 para peso corporal, 0,96-0,99 para estatura, 0,75-0,79 para a força de membros superiores, 0,78-0,82 para a força de membros inferiores, 0,77-0,80 para agilidade e 0,79-0,83 para velocidade.

A distribuição dos dados foi analisada pelo teste de Kolmogorov-Smirnov e as variáveis expressas por média e desvio-padrão para as variáveis numéricas. A variação entre a linha de base e 30 anos foi feita com o delta percentual (∆%). A comparação entre as quatro avaliações foi feita pela análise de variância (Anova) com três fatores (sexo, estado nutricional e década), seguida pelo teste de comparação múltipla de Bonferroni.^16^ Sete valores distintos de percentis (5, 10, 25, 50, 75, 90 e 95) foram calculados em cada década, analisados de acordo com sexo para cada uma das variáveis analisadas. Os cálculos foram feitos com o *software*Statistical Package for the Social Sciences (SPSS) versão 18.0 e foi considerado *p*<0,01.^16^

## Resultados

A proporção de escolares do sexo masculino classificados pelo estado nutricional conforme a década foi: 1978-1980: 27,2% eutróficos e 22,3% com excesso de peso; 1988-1990: 35,8% eutróficos e 20,5% com excesso de peso; 1998-2000: 41,6% eutróficos e 14,9% com excesso de peso; 2008-2010: 43,8% eutróficos e 15,2% com excesso de peso. O número de escolares eutróficos foi maior do que aqueles com excesso de peso em todas as avaliações. Já para o sexo feminino, a proporção foi: 1978-1980 linha de base: 22,8% eutróficas e 27,7% com excesso de peso; 1988-1990: 20,2% eutróficas e 23,5% com excesso de peso; 1998-2000: 24,6% eutróficas e 18,9% excesso de peso; 2008 -2010: 21,6% eutróficas e 19,4% excesso de peso. O número de escolares eutróficas foi maior somente nas avaliações de 20 e 30 anos.^10^ A Tabela 1 apresenta a caracterização (sexo, estado nutricional e antropometria) da amostra de acordo com os quatro períodos de avaliações.

Nos meninos eutróficos, as médias dos valores da força de membros superiores diminuíram significativamente após 30 anos. Já para a agilidade, as médias aumentaram quando se comparou 2008-2010 *vs*. 1978-1980. Não houve diferenças significativas entre os períodos quanto às de força de membros inferiores e velocidade (Tabela 2). Nos meninos com excesso de peso, a força de membros superiores mostrou média estatisticamente superior em 1978-1980, quando comparado com 2008-2010. Não houve diferenças significativas entre os períodos de avaliação de força de membros inferiores, agilidade e velocidade (Tabela 2).

**Tabela 2:** Comparação das variáveis de aptidão física de acordo com sexo e estado nutricional de escolares de Ilhabela em 30 anos

	1978‐1980	1988‐1990	1998‐2000	2008 ‐2010	*p* a	Δ%^b^
**Eutrófico**						
*Sexo*						
Masculino	n=51	n=110	n=156	n=184		
Feminino	n=43	n=62	n=92	n=91		

*FMS (kg)*						
Masculino	21,50±6,92^c^	19,02±4,25^c^	18,06±4,55^c^	17,92±3,65	<0,001	‐16,7
Feminino	18,74±4,88	18,72±4,05	16,18±3,60^c^^,^ d	18,16±4,12	<0,001	‐3,2

*FMI (cm)*						
Masculino	24,60±4,64	24,55±4,82	24,47±4,99	22,38±5,92	0,40	‐9,0
Feminino	24,41±3,86	24,41±4,41	23,51±4,76	22,02±4,89	0,47	‐9,8

*Agilidade (segundos)*						
Masculino	12,54±1,28^c^	12,66±1,19	12,69±1,01	12,73±1,07	<0,001	‐1,5
Feminino	13,51±1,27^c^^,^ d	13,71±1,17^d^	12,93±0,84^d^	13,72±1,60^d^	<0,001	‐1,6

*Velocidade (segundos)*						
Masculino	9,49±0,73	9,70±0,83	9,88±0,86	10,10±2,37	0,04	‐6,4
Feminino	9,59±0,66	10,36±0,87^d^	10,36±0,72	10,32±0,83	0,89	‐7,0

**Excesso de peso**						
*Sexo*						
Masculino	n=42	n=63	n=56	n=64		
Feminino	n=52	n=72	n=71	n=82		

*FMS (kg)*						
Masculino	23,75±6,18^c^	20,83±4,24^e^	18,69±5,20^e^	20,07±4,33^e^	<0,001	‐15,5
Feminino	22,60±5,17	20,56±4,96^c^^,^ e	15,73±3,86^c^^,^d^,^ e	20,08±4,35^e^	<0,001	‐12,5

*FMI (cm)*						
Masculino	22,31±4,62	24,10±6,04	22,60±5,90	24,58±4,75	0,27	10,2
Feminino	24,75±5,44	23,09±4,58	21,32±4,32	23,10±5,42	0,14	‐6,7

*Agilidade (segundos)*						
Masculino	13,15±1,02	13,04±1,14	12,82±1,00	13,06±0,89	0,54	0,7
Feminino	13,86±1,40^d^	13,83±1,24^d^	13,13±1,05	13,66±0,97	0,04	1,4

*Velocidade (segundos)*						
Masculino	10,00±0,68	10,05±0,98	10,53±1,20^e^	10,43±1,00	0,13	‐4,3
Feminino	10,10±0,40	10,60±0,82	11,06±1,14^e^	10,92±1,08	0,17	‐7,5

FMS, força de membros superiores; FMI, força de membros inferiores; dados descritos em média ± desvio padrão.

a Anova com três fatores (sexo, estado nutricional e década).

b Δ% (delta percentual): variação entre a linha de base e 30 anos.

c *p*<0,01: para comparação entre linha de base, 10 anos, 20 anos, 30 anos.

d *p*<0,01: para comparação entre meninos e meninas.

e *p*<0,01: para comparação entre eutróficos e excesso de peso.

Nas meninas eutróficas, a força de membros superiores apresentou média superior quando comparado o período de 1998-2000 *vs*. 2008-2010. As médias dos tempos da agilidade aumentaram quando se comparou 2008-2010 *vs*. 1978-1980. Tanto a força de membros inferiores quanto a velocidade não apresentaram diferenças entre os períodos de avaliações (Tabela 2). Nas meninas com excesso de peso, somente as médias da força de membros superiores diminuíram estatisticamente quando comparados os períodos 1988-1990 *vs*. 2008-2010 e 1998-2000 *vs*. 2008-2010 (Tabela 2).

Em relação à força de membros superiores, os meninos com excesso de peso tiveram desempenho melhor do que os eutróficos nos períodos 1988-1990, 1998-2000 e 2008-2010. Já quanto à velocidade, os eutróficos do sexo masculino tiveram desempenho significativamente melhor do que aqueles com excesso de peso somente no período de 1998-2000. Meninos eutróficos e com excesso de peso tiveram resultados semelhantes na força de membros inferiores e agilidade em todos os períodos analisados (Tabela 2).

As meninas com excesso de peso tiveram desempenho superior quanto à força de membros superiores, comparadas com as eutróficas, nos períodos de 1988-1990, 1998-2000 e 2008-2010. Já para a velocidade, as meninas eutróficas tiveram desempenho significativamente melhor do que aquelas com excesso de peso somente no período de 1998-2000 (Tabela 2).

Em termos de comparação entre os sexos, no que se refere à força de membros superiores, as meninas eutróficas e com excesso de peso tiveram um desempenho pior do que os meninos eutróficos e com excesso de peso somente em 1998-2000. Na agilidade, meninas eutróficas tiveram um desempenho pior do que os meninos eutróficos em todos os períodos de avaliações. Já para as meninas com excesso de peso comparadas com os meninos com excesso de peso, a diferença entre as médias ocorreu somente em 1978-1980 e em 1988-1990. Quanto às médias da força de membros inferiores e à velocidade, não foram encontradas diferenças estatísticas entre os sexos (Tabela 2).

Independentemente da classificação do estado nutricional, as Figuras 1 (sexo masculino) e 2 (sexo feminino) ilustram os valores dos percentis para cada variável de aptidão física e as suas respectivas mudanças ao longo dos anos. No sexo masculino, as médias dos valores dos percentis (50, 75, 90 e 95) da força de membros superiores diminuíram significativamente quando se comparou 1978-1980 com 1988-1990, 1998-2000 e 2008-2010 (Figura 1). Já no sexo feminino, não houve diferenças significativas dos percentis entre os períodos de avaliações para todas as variáveis analisadas (Figura 2).

**Figura 1:** Valores de percentis para cada variável de aptidão física independentemente do estado nutricional em 30 anos, no sexo masculino. FMS, força de membros superiores; FMI, força de membros inferiores; *p<0,01: para comparações entre linha de base com 10 anos, 20 anos e 30 anos.

**Figura 2:** Valores de percentis para cada variável de aptidão física independentemente do estado nutricional em 30 anos, no sexo feminino. FMS, força de membros superiores; FMI, força de membros inferiores.

## Discussão

Nas crianças eutróficas de ambos os sexos, observou-se redução significativa nas médias da força de membros superiores e do tempo da agilidade. Já naqueles com excesso de peso, de ambos os sexos, houve diminuição significativa apenas na força de membros superiores ao longo de 30 anos. Esses resultados parecem indicar que os indicadores de aptidão física de escolares de Ilhabela pioraram nos últimos anos, embora não tenha havido evidência de um aumento da obesidade infantil nessa amostra ao longo dos anos, diferentemente da mudança nutricional de desnutrição para excesso de peso que afetou a população brasileira no mesmo período em que nossos dados foram coletados.^17^ Embora Ilhabela tenha exibido um notável desenvolvimento econômico nas últimas décadas, com o aumento das taxas de população e de urbanização, é importante notar que o estado nutricional permaneceu inalterado entre as crianças estudadas nessa faixa etária. Parece provável, portanto, que os escolares observados neste estudo não seguiram o perfil de transição nutricional dos escolares brasileiros no mesmo período avaliado. A Pesquisa de Orçamentos Familiares^17^ mostrou um aumento de mais de três vezes, de 9,8% para 33,4%, na proporção de crianças brasileiras com excesso de peso entre 1975 e 2009.

Dollman et al.^18^ encontraram um declínio anual na aptidão física em escolares da Austrália e corroboraram os achados do presente estudo. A redução da aptidão física foi observada não só naqueles com excesso de peso, mas também nos eutróficos.^19^ Nossos resultados mostraram pior desempenho nos escolares com excesso de peso do que nos eutróficos somente no que se refere à velocidade. Por outro lado, aqueles com excesso de peso tiveram melhor desempenho na força de membros superiores. Kim et al. 20analisaram escolares dos Estados Unidos e mostraram piores resultados de aptidão física em escolares obesos do que nos eutróficos em testes de corrida. Em ambos os sexos, nossos resultados não encontraram alterações na força de membros inferiores e agilidade, quando comparados escolares eutróficos com aqueles portadores de excesso de peso. Por exemplo, Dollman et al.^18^ encontraram aumento no tempo de corrida de 50 metros em 0,1-0,2% por ano durante 12 anos. Nossos resultados encontraram uma redução na velocidade de 0,24% e 0,20% ao ano em escolares eutróficos e com excesso de peso, respectivamente.

As mudanças da aptidão física ao longo dos anos têm sido atribuídas em grande parte às mudanças na atividade física.^21^Um estudo feito por Artero et al.^22^indicou que crianças com excesso de peso podem executar testes de aptidão física tão bem ou até melhor do que escolares eutróficos. No presente estudo essa tendência foi encontrada no teste de força de membros superiores, especialmente nos meninos.

Outros resultados mostraram que escolares com melhor aptidão física têm menor chance de ter excesso de peso do que aqueles com pior desempenho de aptidão física.^13^^,^ 23 Por exemplo, Shang et al.^23^ revelaram que escolares com excesso de peso tiveram um pior desempenho em aptidão física em comparação com os eutróficos. Resultados comparáveis com os de um estudo transversal em adolescentes da República das Seychelles por Bovet et al.,^13^ que relataram uma relação inversa entre a aptidão física e peso corporal.

Os escolares com excesso de peso são mais sedentários e fazem menos atividade física moderada a vigorosa do que os eutróficos.^24^ Em uma revisão sistemática, foi estabelecida alta correlação entre inatividade física e composição corporal desfavorável e baixa aptidão física. Todos os 232 estudos revisados relacionaram sedentarismo com fatores de risco para DCV e aumento de riscos para a saúde.^25^

De acordo com o modelo estabelecido pela Tomkinson e Olds,^5^ fatores associados a mudanças seculares são causados por um conjunto de fatores sociais, comportamentais, físicos, psicossociais e fisiológicos. Esses padrões podem ser explicados por maiores taxas de ingestão calórica e redução do gasto de energia, associados a um melhor acesso à tecnologia.

Na análise dos dados de acordo com o percentil, década após década, houve variações e reduções consideráveis nos valores de aptidão física, especialmente na força de membros superiores dos meninos. Por exemplo, observando-se o percentil 95 para o sexo masculino, a força de membros superiores foi de 33 kg em 1978-1980 e 24 kg 30 anos após. Tais mudanças também são relatadas em outro estudo.^26^

Quanto ao sexo, os dados apresentados evidenciam que as meninas tiveram um pior desempenho do que os meninos. Pesquisas feitas ao longo dos anos em países desenvolvidos (Espanha e Holanda) mostraram resultados similares aos aqui encontrados tanto em aptidão como em atividade física.^27^^,^ 28

A composição corporal, a aptidão física e os níveis de atividade física estão fortemente associados com doenças cardiovasculares e mortalidade.^25^^,^ 29 Portanto, a identificação de grupos de risco é essencial para o desenvolvimento de estratégias de intervenção. Mudanças no estilo de vida e manutenção da atividade física regular por meio de iniciativas dos pais e de intervenções de apoio social são estratégias importantes para combater a obesidade infantil e inatividade física.^30^ No entanto, outros estudos prospectivos são necessários para determinar a relação de causa e efeito entre atividade física, aptidão física e peso corporal, uma vez que alguns autores encontraram uma relação inversa entre peso corporal e aptidão física.^13^

As avaliações transversais do presente estudo não permitem estabelecer uma relação causa e efeito. A amostra por conveniência pode ter selecionado as crianças que estavam mais interessadas em fazer os testes de aptidão física e limitado a generalização dos resultados obtidos. Apesar disso, essas limitações não escondem uma tendência de diminuição das variáveis de aptidão física que afeta escolares de países em desenvolvimento. Os resultados obtidos podem ser aplicáveis em crianças brasileiras com as mesmas características. No entanto, eles precisam ser interpretados com cautela, mesmo que a literatura internacional tenha encontrado diminuição das variáveis de aptidão física ao longo dos anos em amostras representativas, fato diferente do atual estudo.^18^^,^ 21 Por outro lado, este é o primeiro estudo brasileiro a apresentar o comportamento das variáveis neuromotoras conforme o estado nutricional de escolares ao longo de 30 anos.

O presente estudo mostrou que a aptidão física de escolares de 10 e 11 anos de ambos os sexos apresentou uma redução significativa ao longo de 30 anos. O declínio nos padrões de aptidão física foi maior em escolares eutróficos (força de membros superiores e agilidade) do que naqueles com excesso de peso. Este estudo enfatiza a complexidade dos padrões de aptidão física dos escolares e seu desenvolvimento ao longo do tempo, uma vez que o estado nutricional pode impactar de maneira distinta esse desfecho ao longo dos anos. Outras estratégias de pesquisa devem ser exploradas para explicar a aptidão física e sua relação com o estado nutricional ao longo dos anos em crianças.
